# MBW complexes impinge on anthocyanidin reductase gene regulation for proanthocyanidin biosynthesis in persimmon fruit

**DOI:** 10.1038/s41598-020-60635-w

**Published:** 2020-02-26

**Authors:** Francisco Gil-Muñoz, Jesús A. Sánchez-Navarro, Cristina Besada, Alejandra Salvador, María Luisa Badenes, María del Mar Naval, Gabino Ríos

**Affiliations:** 1 Instituto Valenciano de Investigaciones Agrarias (IVIA), E-46113 Moncada, Valencia, Spain; 2 Instituto de Biología Molecular y Celular de Plantas, Universidad Politécnica de Valencia-CSIC, E-46022, Valencia, Spain

**Keywords:** Plant development, Fruiting, Secondary metabolism, Agricultural genetics, Plant breeding

## Abstract

MBW protein complexes containing MYB, bHLH and WD40 repeat factors are known transcriptional regulators of secondary metabolites production such as proanthocyanidins and anthocyanins, and developmental processes such as trichome formation in many plant species. DkMYB2 and DkMYB4 (MYB-type), DkMYC1 (bHLH-type) and DkWDR1 (WD40-type) factors have been proposed by different authors to take part of persimmon MBW complexes for proanthocyanidin accumulation in immature fruit, leading to its characteristic astringent flavour with important agronomical and ecological effects. We have confirmed the nuclear localization of these proteins and their mutual physical interaction by bimolecular fluorescence complementation analysis. In addition, transient expression of *DkMYB2*, *DkMYB4* and *DkMYC1* cooperatively increase the expression of a persimmon anthocyanidin reductase gene (*ANR*), involved in the biosynthesis of *cis*-flavan-3-ols, the structural units of proanthocyanidin compounds. Collectively, these data support the presence of MBW complexes in persimmon fruit and suggest their coordinated participation in *ANR* regulation for proanthocyanidin production.

## Introduction

Proanthocyanidins (PAs), or condensed tannins, are flavonoid polymers that accumulate in fruits, leaves, seeds and other tissues of many plants, providing protection against pathogens and herbivores. PAs also contribute to fruit flavour and colour and are considered beneficial for human health in virtue of their antioxidant properties, among other salutary attributes^1^. In persimmon (*Diospyros kaki*), the microstructure and accumulation of soluble PAs of the so called tannin cells has been found related to fruit astringent taste^2,3^, potentially affecting fruit palatability for frugivorous animals^4^. Interestingly, soluble tannins are reduced throughout persimmon fruit development and maturation^3,5^, providing a way to channel the action of frugivores when seeds are fully viable and ready for dispersal. In addition to a poorly known mechanism involving the transcriptional repression of PA biosynthetic enzymes^6^, soluble PA content is reduced during fruit ripening by the production of acetaldehyde by seeds, leading to PA insolubilization and the subsequent astringency loss^7,8^. Although the content of soluble tannins becomes undetectable from a sensory point of view at overripening stages, the concomitant loss of fruit firmness importantly limits fruit postharvest life and therefore the commercialization opportunities. To overcome this limitation, the fruit is harvested before overripening and subjected to deastringency postharvest treatments to remove astringency while maintaining high firmness^9^. Most of deastringency methods in persimmon are based on maintaining the fruit under anaerobic conditions or exposing them to products that induce anaerobic respiration. Under these conditions, soluble tannins are polymerized by acetaldehyde accumulated in the flesh^10,11^. Furthermore, natural non-astringent mutants exist into persimmon germplasm collections. Several of these non-astringent cultivars are hypothesized to carry a recessive mutation in a single gene known as *AST*^12,13^, but the molecular function and identity of this gene remain unknown. Postharvest treatments of astringent varieties improve the postharvest life and the organoleptic quality of treated fruit, however these treatments represent an important production cost and are often a challenge for new varieties or stressed orchards^14^. Thus, studying PAs biosynthesis and metabolism in persimmon fruit may help to better understand the different mechanisms employed by plants to drive frugivore-dependent dispersal of seeds, and to improve deastringency treatments and crop management with the aim to reduce costs and increase the sustainability of persimmon production.

The pathway of PAs biosynthesis has been genetically dissected by the analysis of different seed mutants in *Arabidopsis thaliana*, barley and maize among other species^15^. PAs are formed by condensation of *trans*- and *cis*-flavan-3-ols units, synthesized respectively by stereospecific leucoanthocyanidin reductase (LAR) and anthocyanidin reductase (ANR) enzymes^16,17^. These PA biosynthetic activities and genes are essentially conserved in persimmon, with some regulatory particularities^18^. In persimmon fruit, *ANR* gene is much more expressed than its counterpart *LAR*, consistent with the higher content of *cis*-flavan-3-ols stereoisomers in PA composition^19^. In addition, *ANR* is strongly repressed in advanced steps of fruit maturation, concomitantly with tannin decrease, which points to a role of *ANR* as a major integrative target of PA regulatory pathways in persimmon.

In *Arabidopsis*, regulation of the *ANR* orthologous gene *BANYULS* (*BAN*) and PA accumulation in seed coat requires the concerted action of *TRANSPARENT TESTA2* (*TT2*), *TRANSPARENT TESTA8* (*TT8*) and *TRANSPARENT TESTA GLABRA1* (*TTG1*), encoding respectively a R2R3-MYB transcription factor, a basic helix-loop-helix (bHLH) transcription factor and a WD40-repeat (WDR) protein^20^. These regulatory factors form a ternary complex named MYB-bHLH-WD40 (MBW) that may invoke the participation of alternative MYB and bHLH components for the regulation of particular steps of PA and anthocyanin biosynthetic pathways^21,22^. MBW complexes also contribute to PAs production in edible fruits of crop plants such as grapevine^23^, strawberry^24^, apple^25^ and persimmon^26^.

In persimmon, *DkMYB2* and *DkMYB4* genes cause an altered pattern of PAs accumulation and expression of biosynthetic enzymes when misexpressed, and increase *ANR* promoter transcriptional activity in transient reporter assays when combined with a heterologous bHLH factor from *Arabidopsis*^27,28^. Interestingly, DkMYB2 and DkMYB4 specifically recognize different MYB-binding cis-elements by electrophoretic mobility shift assays, arguing for certain degree of subfunctionalization^28^. On the other hand, a persimmon bHLH gene named *DkMYC1* is underexpressed in non-astringent cultivars, following an expression pattern in fruit similar to *DkMYB4*^29^. DkMYC1 protein interacts with DkMYB2 and DkMYB4, and these, in turn, interact with the WD40-repeat protein DkWDR1 by two-hybrid analysis, suggesting the conserved participation of MBW complexes in PA synthesis regulation in persimmon fruit^26^. In this study, we go further on the cytological and molecular characterization of MBW complex in persimmon by approaching the subcellular localization, protein interaction and transcriptional regulation effects of these MYB (DkMYB2 and DkMYB4), bHLH (DkMYC1) and WD40 (DkWDR1) components.

## Results

### Subcellular localization of MBW complex components

**Figure 1:**
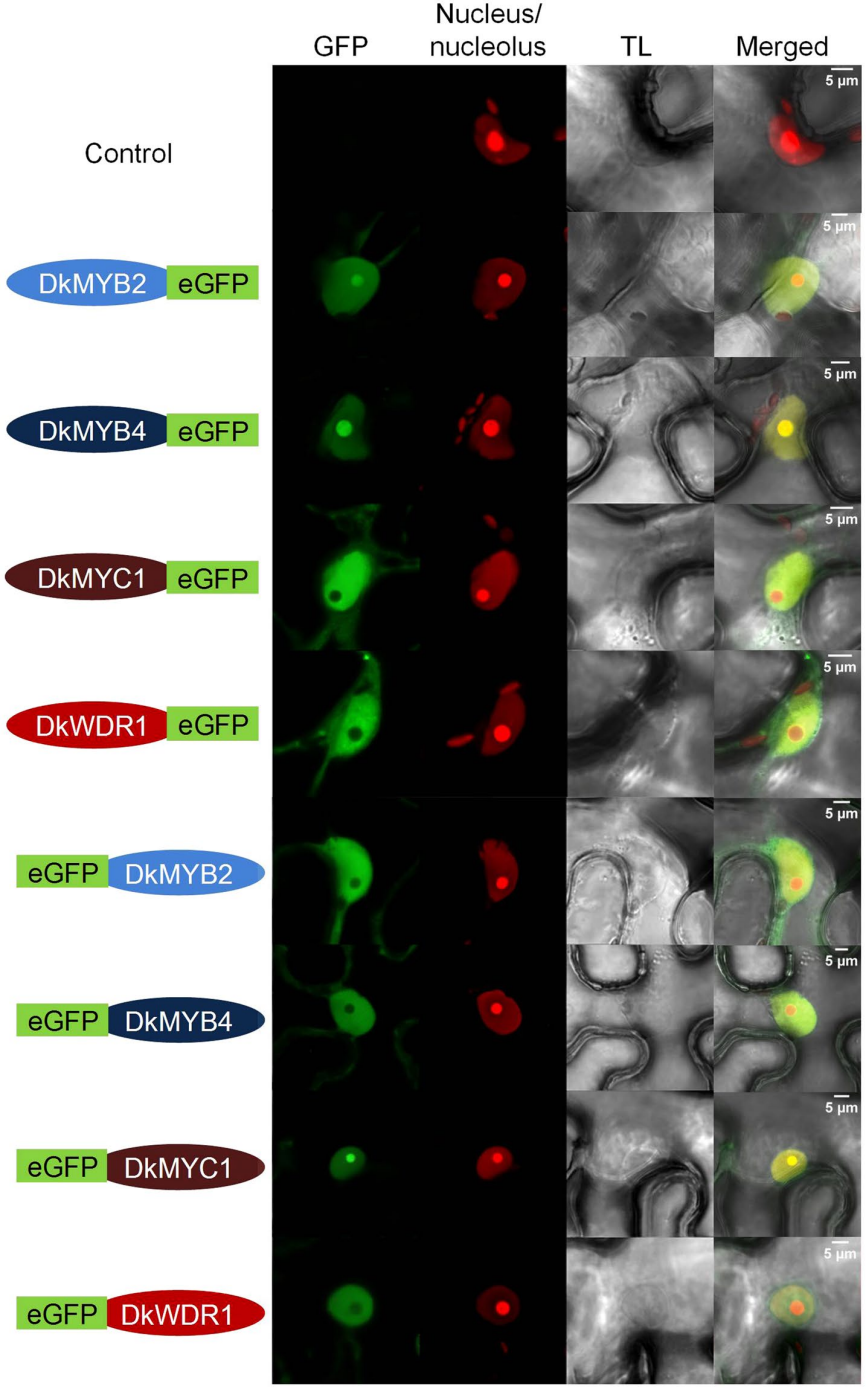
Nuclear localization of MBW factors. *Nicotiana benthamiana* leaves agroinfiltrated with *DkMYB2*, *DkMYB4*, *DkMYC1* and *DkWDR1* constructs containing Ct (right green label) or Nt (left green label) eGFP tags, were co-expressed with nucleus/nucleolus markers. The green (GFP), red (nucleus/nucleolus marker), transmitted light (TL) channels and merged images are shown in the figure. The fluorescent signals were visualized at 72 hours post-infiltration. Scale bars are shown in merged images.

As transcription factors belonging to a hypothetical regulatory protein complex, DkMYB2, DkMYB4, DkMYC1 and DkWDR1 are expected to co-localize temporarily in the cell nucleus where they interact and perform their regulatory role within the framework of a developmental programme. The subcellular localization of these four putative MBW components in persimmon has been elucidated by transient expression in *Nicotiana benthamiana* leaves of the four corresponding genes fused to enhanced green fluorescent protein (eGFP) gene. According to the localization of specific nucleus and nucleolus markers, DkMYB2, DkMYB4 and DkMYC1 show differential nucleus/nucleolus partitioning when eGFP fusion is either at the N-terminal (Nt) or C-terminal (Ct), suggesting that eGFP position affects protein targeting (Fig. 1). Protein fusions with higher abundance in the nucleolus are DkMYB2-eGFP, DkMYB4-eGFP and eGFP-DkMYC1, whereas DkWDR1-eGFP shows appreciable presence in the cytoplasm in spite of its predominant localization in the nucleus. Overall, these four transcription factors show preferential localization in the nuclear compartment, and consequently physical interactions at the protein level occur most plausibly in the nucleus.

### *In vivo* interaction of DkMYB2, DkMYB4, DkMYC1 and DkWDR1

**Figure 2:**
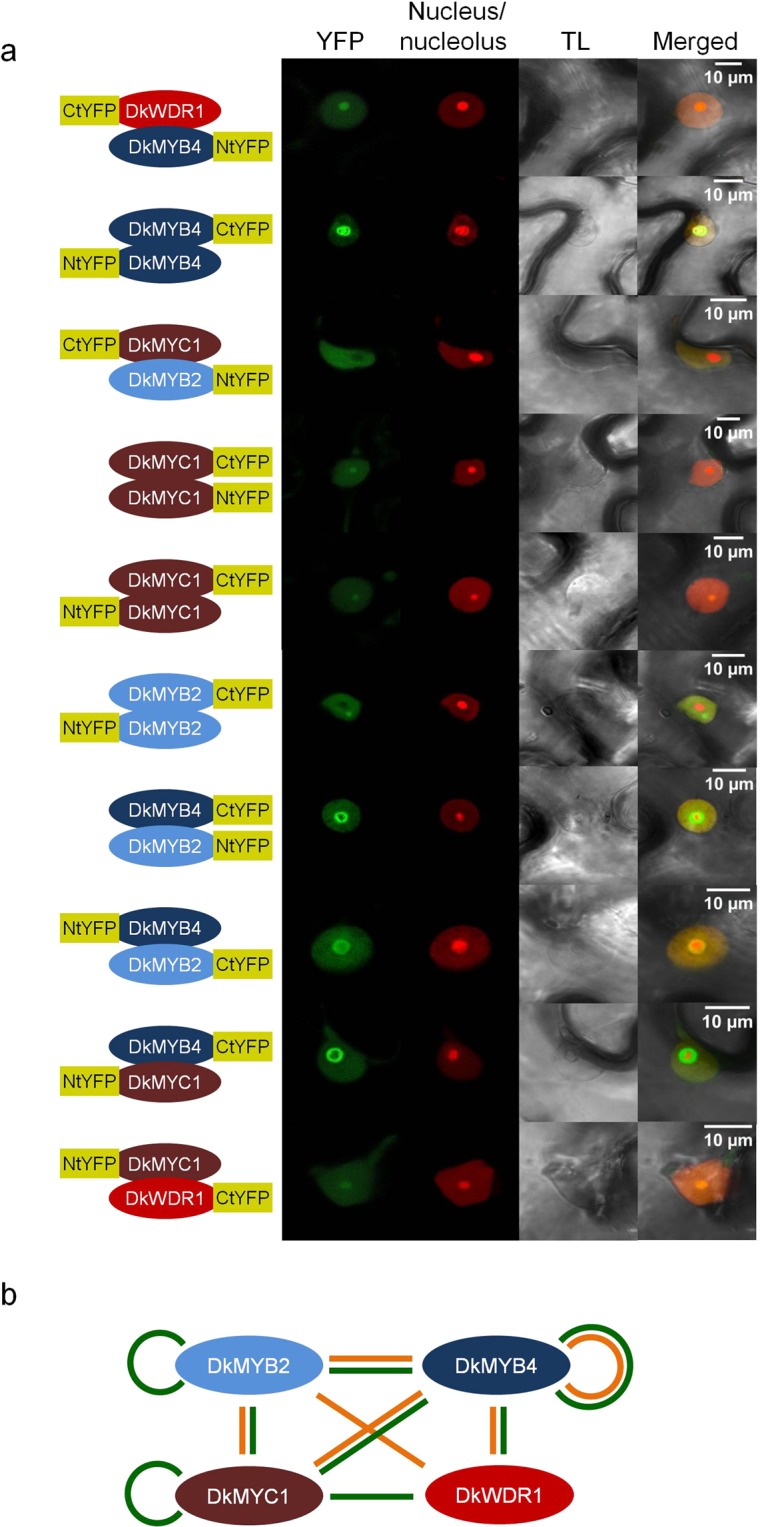
Protein interactions of MBW factors by bimolecular fluorescence complementation assays (BiFC). *N. benthamiana leaves* agroinfiltrated with different combinations of MBW factors fused to NtYFP or CtYFP peptides in Ct (right yellow label) or Nt (left yellow label) positions together with nucleus/nucleolus markers (**a**). The green reconstituted fluorescence (YFP), red (nucleus/nucleolus marker), transmitted light (TL) channels and merged images are shown in the figure. A diagram of previous yeast two-hybrid (orange lines) and positive BiFC interactions obtained in this study (green lines) (**b**). The fluorescent signals were visualized at 72 hours post-infiltration. Scale bars are shown in merged images.

Physical protein interactions among members of MBW complex involved in PAs accumulation in persimmon fruit has been only previously tested by the two-hybrid system in the yeast model, however no additional *in planta* evidences have been obtained on the formation of this complex. To asses this issue, we have assayed pair-wise interactions between DkMYB2, DkMYB4, DkMYC1 and DkWDR1 factors by bimolecular fluorescence complementation (BiFC) and transient expression in *N. benthamiana*. The Nt and Ct fragments of the yellow fluorescent protein (YFP), required for reassembling of the fluorescent reporter, have been tested on both Nt and Ct sides of each transcription factor. Positive protein interactions by BiFC are shown in Fig. 2a. According to these results, DkMYB4 and DkMYC1 are able to interact with the rest of the factors and with themselves, and DkWDR1 does not reconstitute YFP fluorescence when paired with DkMYB2 and itself. This reproduces previous yeast two-hybrid results with few exceptions (Fig. 2b). Particularly, DkMYC1 homodimerization was not observed, and DkMYB2 self-interaction was not assayed due to autoactivation issues in former yeast-two hybrid experiments. Moreover, DkMYB2-DkWDR1 and DkMYC1-DkWDR1 interactions have been exclusively detected by yeast-two hybrid and BiFC analysis, respectively. A western analysis of transiently transformed leaves confirms that *DkWDR1* and *DkMYB2* were successfully co-expressed in different construct combinations and hence, the absent interaction of DkWDR1 with itself and DkMYB2 are not due to deficient protein synthesis or accumulation (Supplementary Fig. S1). Overall, these data support the biochemical ability of these factors to associate in a putative MBW complex *in planta*.

### *DkANR* expression correlates well with PA accumulation and astringency

**Figure 3:**
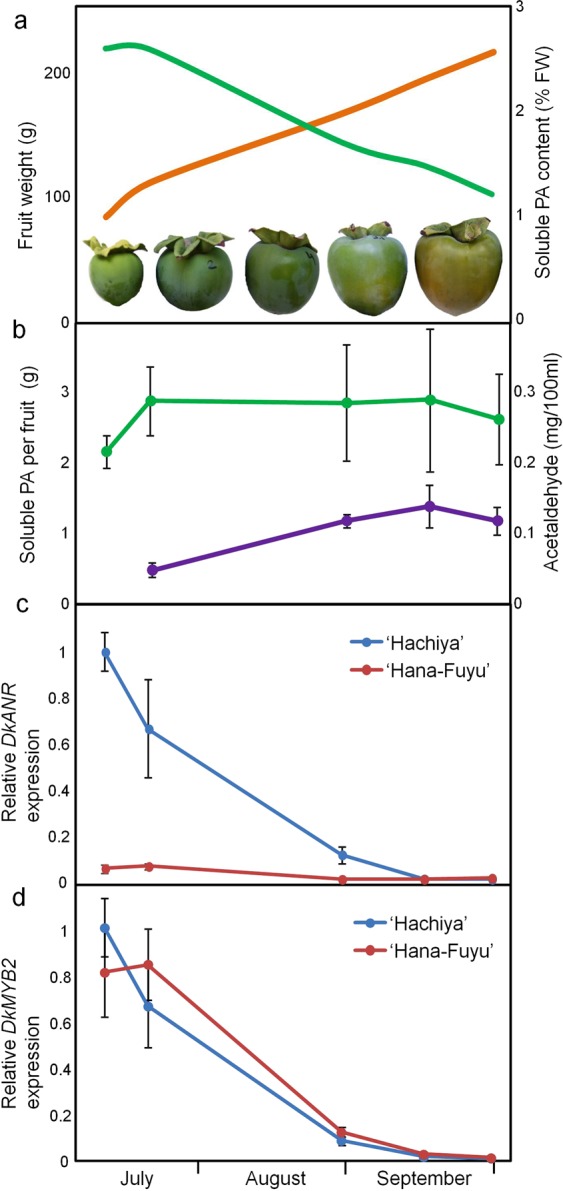
Proanthocyanidin (PA) content and gene expression during fruit development in persimmon. Fruit weight (orange line) and PA relative content (green line) in ‘Hachiya’ cultivar at different fruit development stages (**a**). Acetaldehyde accumulation (violet label) and total estimated PA content per fruit unit (green label) in ‘Hachiya’ (**b**). Relative gene expression of *DkANR* (**c**) and *DkMYB2* (**d**) by qRT-PCR in the fruit samples shown in (**a,b**). The astringent cultivar ‘Hachiya’ (blue label) and the non-astringent cultivar ‘Hana Fuyu’ with the *ast* mutation (red label) have been analyzed. Data are means from four different fruits for anatomical and chemical analysis, and two biological replicates for gene expression. Error bars represent standard deviations.

PA content has been measured at different points of fruit development in ‘Hachiya’ cultivar, starting in July and finishing in September, after external colour change has been initiated and before ripening leads to fruit softening and natural deastringency (Fig. 3a). During that period fruit average weight increases three-fold and the percentage of PA decreases concomitantly (Fig. 3a). Fruit soluble PA content is a balance between PA biosynthesis and insolubilization. PA insolubilization is mediated by acetaldehyde accumulation as a result of ripening and deastringency treatments^10,11^. Acetaldehyde content in these ‘Hachiya’ fruit samples reaches values around 0.1 mg per 100 ml of juice (Fig. 3b), which is by far lower than acetaldehyde produced in stored fruit and fruit treated for deastringency^3,30^. Thus, PA insolubilization due to acetaldehyde accumulation is not expected to contribute significantly to reduce soluble PA level in our samples, and PA content is mostly dependent on its biosynthesis rate. When representing the total estimated amount of PAs per fruit instead of its relative percentage, PA amount remains almost unchanged during the whole interval, with the exception of an initial increase in July samples (Fig. 3b). As we consider that PA reduction by acetaldehyde-dependent insolubilization is relatively low, the observed decrease in relative PA content in Fig. 3a must be mostly due to a growth dilution effect, and the rate of PA biosynthesis is expected to be also low in this period.

As anthocyanidin reductase encoded by *ANR* gene has been postulated to perform a key role in PA biosynthesis, we have measured *DkANR* expression in ‘Hachiya’ fruit samples by qRT-PCR. *DkANR* transcript sharply decreases until a 0.01-fold change during the whole period (Fig. 3c), which cannot be explained by just growth dilution effects. On the contrary, it indicates a strong transcriptional repression in advanced developmental stages. The higher expression of *DkANR* in initial samples is in close agreement with the concurrent increase in total PA content per fruit (Fig. 3b). Then PA content remains steady in concordance with *DkANR* down-regulation (Fig. 3b,c). Such a positive correlation between *DkANR* expression and astringent-responsible PAs is confirmed in the low-PA non-astringent cultivar ‘Hana Fuyu’, which shows a constantly low *DkANR* expression during fruit development stages (Fig. 3c).

Other PA biosynthetic regulators, such as *DkMYB4* and *DkMYC1*, reproduce well this low expression profile in ‘Hana Fuyu’ and other non-astringent cultivars, as shown in previous studies^26,27,29^. However, *DkMYB2* relative expression decays in a similar way in both astringent and non-astringent cultivars (Fig. 3d), suggesting a common mechanism involving *AST* locus-dependent regulation of *DkMYB4* and *DkMYC1*, with no impact on *DkMYB2* expression.

### Effect of MBW factors on the activity of *ANR* promoter

Based on PA-linked expression of these genes, we have cloned three DNA fragments (1.2–1.4 kb) of the promoter and 5′ UTR of *DkMYB4*, *DkMYC1* and the *D. lotus ANR*, in the pGreenII-0800-LUC vector. These vectors synthesize luciferase reporter (LUC) under the action of our selected promoters, in order to test the regulatory effect of individual and combined MBW factors on the activity of these promoters by a dual luciferase assay, using expression of the REN reporter gene as internal reference.

**Figure 4:**
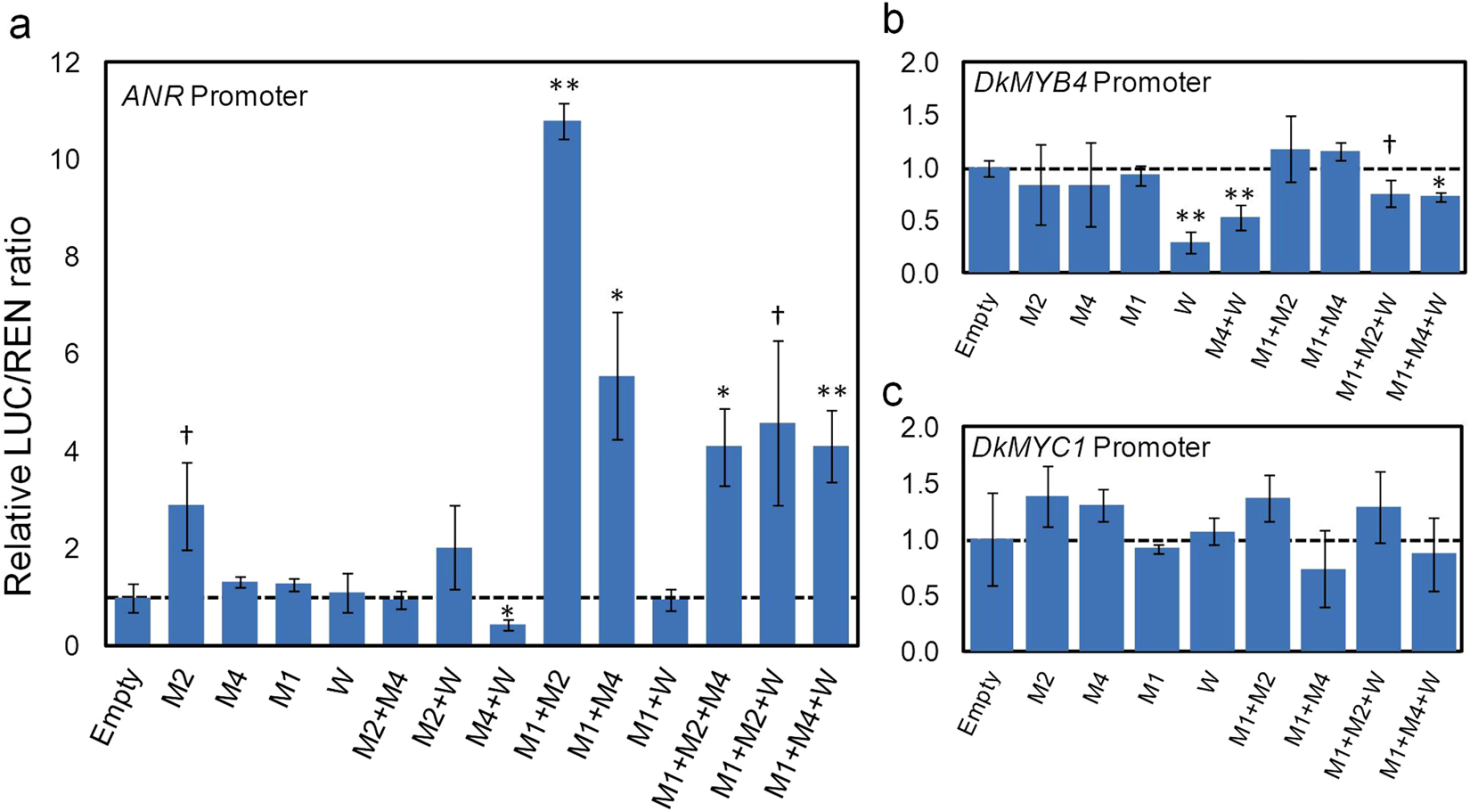
Analysis of the transcriptional activity of relevant PA biosynthesis regulatory promoters by dual luciferase assay. The LUC/REN ratio of *N. benthamiana* cells agroinfiltrated with *ANR* (**a**), *DkMYB4* (**b**) and *DkMYC1* (**c**) promoters driving the LUC gene reporter, and regulatory genes *DkMYB2* (M2), *DkMYB4* (M4), *DkMYC1* (M1) and *DkWDR1* (W) under different combinations, was made relative to mock agroinfiltrations with pGreenII-62-SK (value of 1 labelled with a discontinuous line). Data are means from three replicates with error bars represent standard deviations. Symbols indicate statistical differences with respect to empty sample determined by Student’s *t* test (^†^*P* < 0.1, *P < 0.05, **P < 0.01).

*DkMYB2* transient expression in *N. benthamiana* leaves increases *ANR* promoter transcriptional activity three-fold, whereas *DkMYB4*, *DkMYC1* and *DkWDR1* do not modify it significantly (Fig. 4a). Interestingly, any combination of two or three elements containing both *DkMYC1* and a MYB gene (*DkMYB2* or *DkMYB4*), strongly increases LUC/REN ratio, being highest when *DkMYC1* and *DkMYB2* are co-expressed. On the contrary, *DkWDR1* does not improve *ANR* promoter expression under any gene combination.

As *DkMYB4* and *DkMYC1* genes are similarly down-regulated during fruit development concomitantly with PA reduction, and are differentially expressed in a non-astringent cultivar^26,27,29^, their promoters have been also cloned and tested by dual luciferase assays in *N. benthamiana*. MBW factors assayed in the experiment do not increase the activity of *DkMYB4* nor *DkMYC1* promoters, however *DkWDR1* transient expression associates with a significant decrease in LUC expression driven by *DkMYB4* promoter (Fig. 4b,c).

## Discussion

DkMYB2, DkMYB4, DkMYC1 and DkWDR1 have been postulated to co-regulate the expression of PA biosynthesis genes in persimmon as a complex^26^, and hence a coordinated nuclear co-localization of them is expected. Related components of MBW complexes in other species have been found located in the nucleus (TT2)^31^, or partitioned in nucleus and cytoplasm (TTG1 and VvMYC1)^23,32^. In this study, DkMYB2, DkMYB4, DkMYC1 and DkWDR1 have been mostly localized in the cell nucleus, but DkMYB2, DkMYB4, DkMYC1 show a differential nucleus-nucleolus partitioning depending on the Nt or Ct position of the eGFP fusion. To our knowledge, this has not been previously observed in other MBW factors, although in most of these cases, only one Nt or Ct fusion is assayed. Indeed, a systematic approach in yeast has shown that a high percentage of proteins display different subcellular localization when GFP is tagged at either the Nt or Ct^33^.

Transient expression in *N. benthamiana* and BiFC analysis support the ability of DkMYB2, DkMYB4, DkMYC1 and DkWDR1 proteins to interact with each other *in vivo*, which in fact reinforces previous two-hybrid data in the yeast *Saccharomyces cerevisiae*^26^. The only combinatorial interactions not confirmed by BiFC are DkMYB2-DkWDR1 and DkWDR1 with itself. DkMYB2-DkWDR1 interaction was observed in a previous two-hybrid study, thus only homodimerization of DkWDR1 is not sustained on experimental evidences. These BiFC and yeast two-hybrid interaction data are compatible with a multitude of possible combinations and sizes of the complex, which presumably enable a high degree of functional and regulatory versatility. Physical interactions among MBW factors involved in PA production have been also verified in *Arabidopsis*^20^, grapevine^23^, strawberry^24^, and tea plant^34^ among other PA and flavonoid biosynthesis complexes.

**Figure 5:**
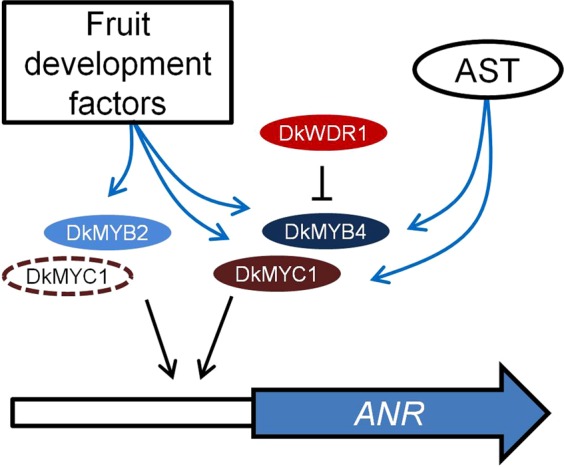
Diagram of regulatory factors affecting *ANR* expression. *ANR* coding gene is represented as a wide blue arrow, and its promoter as a contiguous white rectangle. Regulatory proteins are elliptic forms. The discontinuous ellipse of DkMYC1 indicates it is dispensable for the positive effect of DkMYB2. The transcriptional effect is labelled as a black arrow (inductive) or a black line ended in a perpendicular bar (repressive). Fruit development-dependent factors and AST locus modify the expression of MYB and bHLH factors by a yet unknown mechanism (blue arrows).

These BiFC and subcellular localization results strongly support the formation *in vivo* of protein complexes comprising at least several of these factors, but conclusive functional evidences about their coordinated recruitment to modify the expression of PA biosynthetic genes in persimmon fruit are scarce. Anthocyanidin reductase is the main enzyme specifically involved in PA production in persimmon, and its coding *ANR* gene is considered a major target of transcriptional regulation^19^, being consequently a proper candidate gene for studying PA biosynthesis regulation. Previous dual luciferase assays in *N. benthamiana* have shown that *DkMYB2* and *DkMYB4* increase the activity of *ANR* promoter when co-expressed with *Arabidopsis AtEGL3* gene coding for a bHLH protein involved in regulation of the flavonoid pathway, but not when expressed individually^28^. On the contrary, in our hands, *DkMYB2* is able to increase *ANR* promoter activity three-fold in the absence of other factors, which suggests that DkMYB2 does not require a complete MBW complex to enhance, at some level, *ANR* expression and consequently improve PA production (Fig. 5). Interestingly, the ectopic expression of *DkMYB2* in kiwifruit calluses induces PA accumulation without additional MBW components^28^. *DkMYB2* expression is regulated by fruit maturation factors in persimmon that markedly reduce it in advanced stages of development, similarly to *ANR*, *DkMYB4* and *DkMYC1*, but in contrast to these genes it seems not to be impaired in *ast* non-astringent mutants. Unexpectedly, *DkMYB2* has only a minor contribution to PA accumulation in these mutants, which is most likely due to its low expression level in fruit in comparison with *DkMYB4*^27^.

We have employed for the first time the persimmon bHLH (DkMYC1) and WD40 (DkWDR1) components in transient expression assays in combination with MYB factors, for the elucidation of the function of MBW complexes in the expression of PA responsive genes in persimmon fruit. *DkMYC1* does not affect significantly *ANR* expression on its own, but consistently intensifies the effect of *DkMYB2* and *DkMYB4*. In *Arabidopsis*, there is a similar synergistic effect of *TT2* (MYB) and *TT8* (bHLH) on the expression of *BAN* that responds to a stronger cooperative binding of the pair TT2-TT8 to *BAN* promoter^20^. Thus, the low expression of *ANR* gene from early stages of fruit development in the *ast* non-astringent cultivar ‘Hana Fuyu’ (Fig. 3c) seems to be caused by the concomitant defective expression of *DkMYB4* and *DkMYC1* genes in this mutant^26^ (Fig. 5). Contrarily to the positive effect of *TTG1* (WD40) gene overexpression, and the negative effect of *TTG1* silencing on *BAN* expression in *Arabidopsis*^20^, *DkWDR1* expression does not affect *ANR* promoter activity in our transient expression experiments. The strawberry ortholog of TTG1 also increases *Arabidopsis BAN* expression in combination with its bHLH and MYB co-interactors^24^, which suggests the presence of certain functional particularities in persimmon DkWDR1 or perhaps regulatory differences between *ANR* and *BAN* promoters. On the other side, WD40 proteins act as structural platforms for facilitating protein-protein interaction, and consequently its effect on the positive transcriptional activity of the complex could be shaded by the ectopic overexpression of components of the complex and the presence of endogenous similar factors in *N. benthamiana* cells.

As *DkMYB4* and *DkMYC1* show a development and cultivar dependent expression profile highly similar to *ANR*^26,27,29^ (Fig. 3c), we considered plausible a self-regulatory loop in the expression of these genes, similar to the positive feedback mechanism operating in *TT8* from *Arabidopsis*^35^. However, *DkMYB4* and *DkMYC1* promoters are not activated by any MBW element utilized in this study. On the contrary, *DkWDR1* reduces the expression of *DkMYB4* by itself and in combination with MYB genes. This repressive effect of *DkWDR1* resembles the activity of MBW complexes containing MYB proteins showing at the C-terminal end an ethylene-responsive element binding factor-associated amphiphilic repression (EAR) motif^36^. Thus, binding of specific repressive MYB proteins has potentially the ability to turn MBW complex into a repressive factor. In light of these data we cannot discard a repressive role of *DkWDR1* on the expression of *DkMYB4* and other genes, which could depend on the binding of distinct MYB of bHLH elements with specific activating or repressive domains.

## Methods

### Plant material

*Diospyros kaki* Thunb. cvs ‘Hachiya’ (astringent fruit) and ‘Hana Fuyu’ (non-astringent fruit) were grown in an orchard located in Museros (Spain; 39°34′40″N, 0°21′46″W) under standard agricultural practices^37^. Four fruit samples per cultivar were collected at different maturation stages on the following dates in 2011: July 12, July 21, August 30, September 16 and September 30^26^. Soluble tannins were evaluated using the Folin-Denis method^38^, as described previously^39^, and results were expressed as percentage of fresh weight (FW).

### Gene isolation

The *Diospyros kaki* (cv. ‘Hachiya’) genes *DkMYB2* (AB503699.1), *DkMYB4* (KR057233.1), *DkMYC1* (KR057234.1) and *DkWDR1* (KR057229.1) were obtained from pGADT7 plasmids described in a previous study^26^. In order to identify *DkMYB4* and *DkMYC1* promoter sequences, a manual assembly of *D. lotus* cv. Kunsenshi genome reads stored in the Sequence Read Archive (SRA) database (ID: SRP045872) was performed^40^. For amplifying *ANR* promoter, we designed primers (Supplementary Table S1) from the previously published sequence of *D. lotus* gene (AB504523.1). *ANR* promoter amplification was not possible in *D. kaki*, and therefore the *D. lotu*s promoter was used in the analysis. *DkMYB4* and *DkMYC1* promoters were amplified from *D. kaki* cv. ‘Hachiya’ genomic DNA (Supplementary Table S1). The DNA of both *D. kaki* and *D. lotus* was extracted from fresh leaves following a standard CTAB DNA extraction protocol^41^.

### Plasmid construction

For the construction of subcellular localization and BiFC vectors, each of the four genes were amplified (Supplementary Table S1) with a 15 bp target vector residue at the 5′ end needed for recombination with the In-Fusion HD Cloning kit (TAKARA BIO, Otsu, Japan). Fragments were purified and cloned into ampicillin resistant pSK + 35S-eGFP-PoPit vectors^42^ and two cassettes for each protein were made for subcellular localization. Four cassettes for each protein were made with ampicillin resistant pSK + 35S-(N-YFP or C-YFP)-PoPit vector^43^ for BiFC analysis. The fragments containing the expression cassette from pSK vectors were digested with *Hin*dIII and subcloned into the kanamycin resistant pMOG800 vector^44^.

In order to construct the vectors for transient expression, the four genes contained in pGADT7 plasmids^26^ were digested with *Sac*I and *Xho*I to release the insert. The fragments were purified and inserted in a pGreenII-62-SK kanamycin resistant vector^45^, previously linearized with *Sac*I/*Xho*I. The promoter sequences were amplified from vectors using specific primers with restriction enzyme sequences tails at 5′ (Supplementary Table S1). The purified PCR products were digested (*Hin*dIII/*Pst*I for *ANR* and *DkMYC1* promoters and *Hin*dIII/*Nco*I for *DkMYB4* promoter), purified and cloned into linearized pGreenII-0800-LUC kanamycin resistant vector^45^.

All the described vectors were provided by Dr. J. A. Sánchez-Navarro (Instituto de Biología Molecular y Celular de Plantas “Primo Yúfera”, Valencia, Spain). Plasmids containing gene and promoter constructs were finally transferred to *Agrobacterium tumefaciens* strain C58 by electroporation. For pMOG800 vectors, transformation was carried out in bacteria containing the virulence helper plasmid pCH32^46^. All DNA constructions were verified by plasmid DNA sequencing.

### Subcellular localization of MBW complex proteins *in vivo*

To characterize the subcellular localization of the MBW complex components, each protein was fused at either the Nt or the Ct of eGFP and transiently expressed *in planta*. For a better visualization of the fluorescence signal, all proteins were co-expressed with the silencing suppressor HC-Pro protein from the *Tobacco Etch Virus*^43^. *A. tumefaciens* C58 strains were grown overnight in LB media supplemented with kanamycin and rifampicin, at 28 °C. Cultures were centrifuged 5 min at 4,000 × g, and pellets were resuspended in infiltration media (MgCl_2_ 10 mM + MES 10 mM pH 5.6) to an OD_600_ of 0.5 for each construct and an OD_600_ of 0.1 for the HC-Pro. *N. benthamiana* young plants (2 pairs of leaves) were agroinfiltrated as previously described^47^. Plants remained in a greenhouse at 24 °C (day) and 18 °C (night) with a 16 h light photoperiod. Three days after infiltration, leaf samples were collected and mounted in a microscope slide with a drop of water. Observation of the fluorescence in the underside epidermis was performed with a LEICA TCS SL confocal microscope (λ_exc_ = 488 nm; λe_m_ = 492–533 nm for eGFP). For the nucleus and nucleolus subcellular colocalization, the proteins were coinfiltrated with cultures (OD_600_ 0.1) expressing the NLS of SV40 large T antigen fused to the red fluorescent protein and the fibrillarin fused to the cherry fluorescent protein, respectively (λ_exc_ = 561 nm; λe_m_ = 588–634 nm).

### Bimolecular fluorescence complementation assays (BiFC)

In the BiFC assay^48^, addressed to characterize the interaction between the components of the hypothetical MBW complex, all the possible two-by-two combinations of homodimers and heterodimers were assayed *in planta*. Chimeric proteins were transiently co-expressed in *N. benthamiana* using *A. tumefaciens* (strain C58) cultures (OD_600_ = 0.4) transformed with the corresponding binary plasmids pMOG800, as previously described^47^. To increase the expression of the different proteins, we included an *A. tumefaciens* culture (OD_600_ = 0.1) expressing the HC-Pro. At 3 days post-infiltration, the fluorescence reconstitution was monitored in the confocal LEICA TCS SL (λ_exc_ = 488 nm; λ_em_ = 492–533 nm).

### Western blot assay

Samples from the BiFC assay were immediately frozen in liquid nitrogen. Frozen leaves (50 mg) were ground and proteins were extracted with 200 µl of Laemmli buffer^49^, boiled for 5 min and centrifuged for 1 min at 15,800 × g to pellet cellular debris. For protein separation, 25 µl of the mixture was subjected to 12% sodium dodecylsulphate‐polyacrylamide gel electrophoresis (SDS‐PAGE). Gels were electrotransferred to nitrocellulose membranes following the manufacturer’s recommendations. Proteins were detected on western blots using an anti‐GFP N‐terminal antibody (SIGMA, St. Louis, MO, USA; cat. no. G1544) for N-terminal yellow fluorescent protein (NtYFP) fusions, and anti‐GFP antibody (ROCHE, Basel, Switzerland; cat. no. 11814460001) for C-terminal YFP (CtYFP) fusions, followed by a secondary peroxidase-labelled antibody and incubation with a chemiluminescence substrate (AMERSHAM, ECLTM Prime Western Blotting Detection Reagent). The chemiluminescence was detected exposing photographic film to the membranes.

### Dual luciferase assay

To determine the effects of the hypothetical MBW protein complex on the promoters of *ANR*, *DkMYB4* and *DkMYC1* genes, a dual luciferase assay was performed. The effect of the homo and heterodimers formed by two and three proteins was assayed by the co-expression of the promoter and protein vectors as previously described in *N. benthamiana* with *A. tumefaciens* strain C58. HC-Pro was also used for enhancing the transient expression of the different proteins. After 3 days, 30 mg of agroinfiltrated leaves from 3 biological replicates, were sampled for each combination. Samples were immediately frozen in liquid nitrogen. For measuring promoter activity, samples were ground to powder and then Firefly (LUC) and Renilla (REN) luciferase activity was measured following the Dual-Luciferase Reporter Assay System (PROMEGA, Madison, WI, USA) with the aid of a PROMEGA GloMax Multi Microplate Reader luminometer. Promoter activity was measured as the quotient between the LUC/REN ratio of promoter plus transcription factors samples and the LUC/REN ratio of promoter without additional factors.

### Isolation of RNA and quantitative real-time RT-PCR (qRT-PCR)

Total RNA was isolated from 150 mg of fruit flesh using a cetyltrimethylammonium bromide (CTAB)-based procedure^50^. Genomic DNA was removed with the RNase-Free DNase Set (QIAGEN, Hilden, Germany) according to manufacturer’s instructions. Purified RNA was reverse transcribed with PrimeScript RT Reagent Kit (TAKARA BIO). qRT-PCR was performed in a StepOnePlus Real-Time PCR System (LIFE TECHNOLOGIES, Carlsbad, CA, USA), using 1–2 µl of 10X diluted cDNA, SYBR premix Ex Taq (Tli RNaseH plus) (TAKARA BIO) and primers shown in Supplementary Table S1, in a total volume of 20 µl. The PCR protocol consisted of 10 min at 95 °C, followed by 40 cycles of 15 s at 95 °C, and 1 min at 60 °C. PCR specificity was confirmed by the presence of a single peak in the dissociation curve and by agarose electrophoresis. We used DkActin as reference gene^26,27^. A relative standard curve procedure was employed for measuring relative expression. Results were the average of two independent biological replicates with 2–3 technical replicates each.

## Supporting Information

Supplementary Information.
