# Supplementary material for: MBW complexes impinge on anthocyanidin reductase gene regulation for proanthocyanidin biosynthesis in persimmon fruit
Source: Sci Rep. 2020 Feb 26;10:3543. doi: 10.1038/s41598-020-60635-w (PMC7044221; doi:10.1038/s41598-020-60635-w)
Supplement: Supplementary file 1 — Supplementary Information. [file 41598_2020_60635_MOESM1_ESM.pdf]

Supplementary information

MBW complexes impinge on anthocyanidin reductase gene regulation for proanthocyanidin biosynthesis in persimmon fruit

Francisco Gil-Muñoz[1], Jesús A. Sánchez-Navarro[2], Cristina Besada[1], Alejandra Salvador[1], María Luisa Badenes[1], María del Mar Naval[1], and Gabino Ríos[1,*]

[1]Instituto Valenciano de Investigaciones Agrarias (IVIA), Moncada, Valencia, Spain
[2]Instituto de Biología Molecular y Celular de Plantas, Universidad Politécnica de Valencia-CSIC, Valencia, Spain

*Corresponding author: Gabino Ríos. Instituto Valenciano de Investigaciones Agrarias (IVIA), Carretera CV-315, Km 10,7 Moncada 46113 (Spain). Phone: (+34) 963424140. e-mail: rios_gab@gva.es

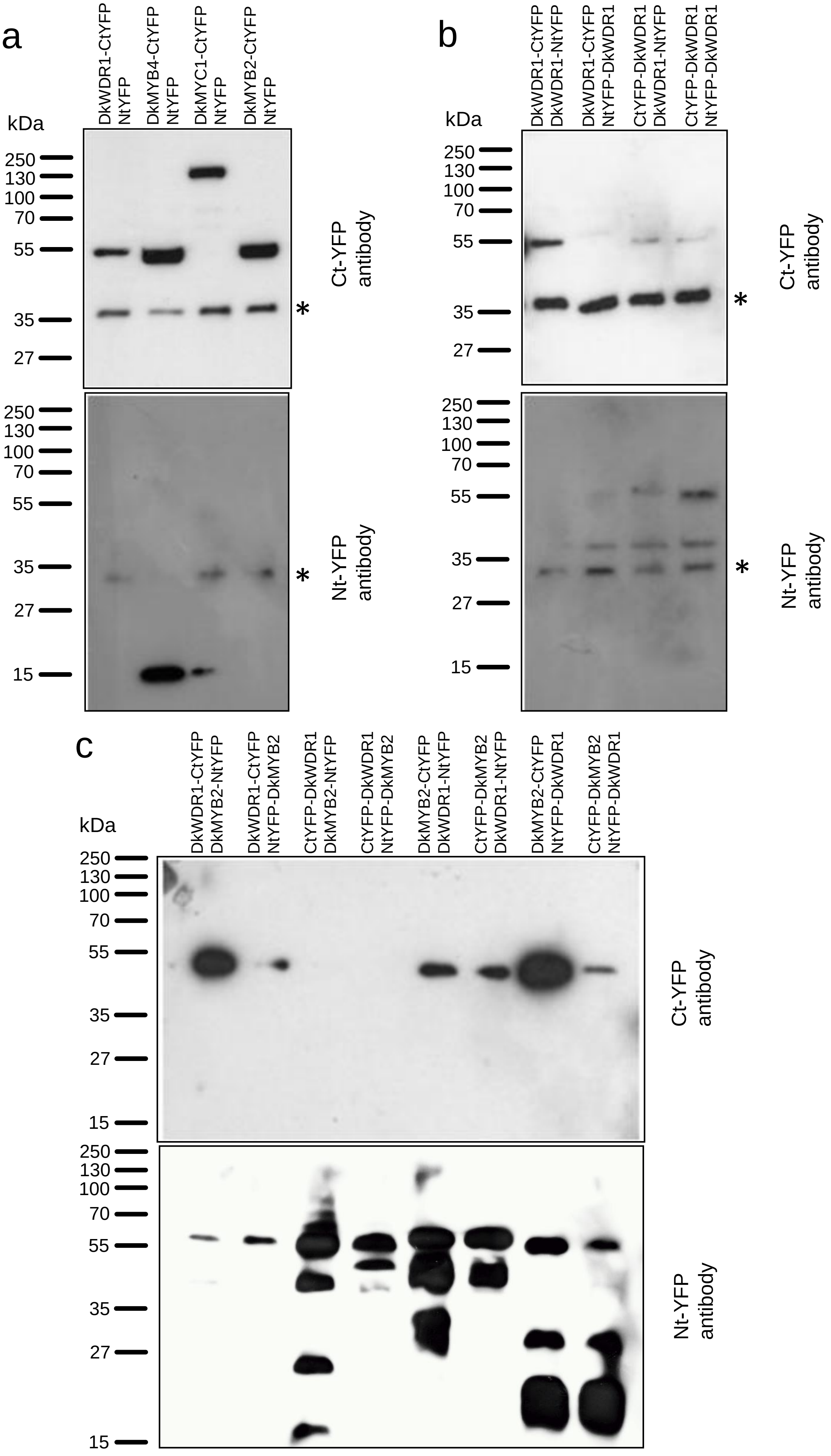

Figure S1. Western blot analysis of BiFC negative interaction combinations. Combinations are DkMYB2, DkMYB4, DkMYC1 and DkWDR1 with NtYFP (a), DkWDR1 with itself (b), and DkWDR1 with DkMYB2 (c). Molecular weight markers are shown on the left. Antibodies are shown on the right. Unspecific bands are labelled (*).

Table S1. Primers used in this work.

	Forward	Reverse	Amplicon length (bp)
Primers used for promoter cloning			
ANR promoter (cloning)	AAAAGTCTTGGGAGTCGTGCCTTC	ACGCTGCCTTGGCTCCTAACG	1415
DkMYB4 promoter (cloning)	AATTCACGGCCATGGAAACGTGGT	CTCTTCTTCTGATATTTGTGACGCAG	1392
DkMYC1 promoter (cloning)	CGTGCGAGCATTCATGAGATGAGA	ACTGTAAGTCCATTGCACCGTCTGA	1268
Subcellular localization and BiFC			
DkMYB2-Fluorescent protein (FP)	AGGATCGGTACCATGGGAAGGAAGCCATGCTGTG	CTGCACCGGCGCTAGCTGCGCCTAAACCATGGGGT	
FP-DkMYB2	GGTGCAGGAGCCATGGGAAGGAAGCCATGCTGTG	TCTAGGGAGCGCTAGCCTAGCCTAAACCATGGGGTTGG	
DkMYB4-FP	AGGATCGGTACCATGGGAAGAGCTCCTTGTTGTTC	CTGCACCGGCGCTAGCGATGAGCAATGATTCAGCAAAGG	
FP-DkMYB4	GGTGCAGGAGCCATGGGAAGAGCTCCTTGTTGTTC	TCTAGGGAGCGCTAGCTCAGATGAGCAATGATTCAGCAAAGG	
DkMYC1-FP	AGGATCGGTACCATGGCCGCTCCGCCTAGTT	CTGCACCGGCGCTAGCATGTATTATCTGATGTATCGCCCTCTTC	
FP-DkMYC1	GGTGCAGGAGCCATGGCCGCTCCGCCTAGTT	TCTAGGGAGCGCTAGCTTAATGTATTATCTGATGTATCGCCCTCTTC	
DkWDR1-FP	AGGATCGGTACCATGGAGAATTCGACCCTAGAG	CTGCACCGGCGCTAGCAACTTTTAGAAGCTGCATTTTGG	
FP-DkWDR1	GGTGCAGGAGCCATGGAGAATTCGACCCTAGAG	TCTAGGGAGCGCTAGCTTAAACTTTTAGAAGCTGCATTTTGG	
Transient expression vector construction			
ANR -Luc	CGGAAGCTTAAAAGTCTTGGGAGTCGTGCCTTCAAC	TATCTGCAGACGCTGCCTTGGCTCCTAACGTTG	
DkMYB4 -Luc	CGCAAGCTTGTATAGCTTGAATAGAATGAAACTTGTAG	TAACCATGGCTCTTCTTCTGATATTTGTGACGCAG	
DkMYC1 -Luc	GCCAAGCTTCGATTTGGGATAATTTCAACTAAGGAGAAT	TATCTGCAGCTACGCCGCTCTGCTAGCTGTCAC	
